# A Quick Analysis Method for Protein Quantification in Oilseed Crops: A Comparison With Standard Protocol

**DOI:** 10.3389/fnut.2022.892695

**Published:** 2022-05-31

**Authors:** Sapna Langyan, Rakesh Bhardwaj, J. Radhamani, Rashmi Yadav, Raj Kumar Gautam, Sanjay Kalia, Ashok Kumar

**Affiliations:** ^1^ICAR-National Bureau of Plant Genetic Resources (NBPGR), New Delhi, India; ^2^Department of Biotechnology (DBT), New Delhi, India

**Keywords:** Kjeldahl method, protein quantification, digestion, distillation, titration, oilseed crops, whole grain, flour

## Abstract

Protein is one of the most abundant substances in plants and plays a major role in human health hence standardization of its analytical quantification method is essential. Various methods for protein quantification exist, such as Kjeldahl, Bradford, Lowry, bicinchoninic acid assay (BCA), Biuret, and total amino acid content methods. These methods are widely applied; however, the development of the rapid and efficient method is the need of the time hence the objective of this research was to analyze and comparing compare the modification of the Kjeldahl method for the determination of protein content in oilseed crops. The study was performed to improve the sample preparation method (processing and digestion) for protein quantification. Generally, the method initially requires homogenization of grains to a fine flour, which involves time and increases the risk of sample cross-contamination and partial loss of oil from the sample during grinding. Moreover at times, it becomes challenging to homogenize oil seeds to fine flour due to high oil content. However, in the present research, the whole grain was digested in place of grounded flour to accomplish quick protein quantification and compared it with the flour matrix of different oil seeds. To further reduce the digestion time and avoid frothing, we have used the modified digestion mixture. The developed method was statistically validated using analysis of variance (ANOVA), Pearson correlation reliability test, paired *T*-test, and different types of plot analysis. The validation of the sample preparation method in protein quantification demonstrated non-significant differences that the protein content from whole grain of all the five oilseed crops shows 100% non-significant results compared with the flour matrix in both the digestion mixtures. The developed novel method could be used to prepare the sample for protein analysis and reduces the overall analysis time while ensuring the accuracy of the results.

## Introduction

The quality of protein and its type is important to determine its overall health impact upon consumption (1, 2). Protein provides energy, and it is also a vital component for other purposes, including enzyme activity and bio-chemicals passage in cellular membranes (3, 4). The accuracy in quantification of protein content is important to further determine its food’s economic value (5, 6). To determine protein’s quantity in food matrices, several methods are used and reported such as Kjeldahl nitrogen estimation, colorimetric assays (Bradford, Lowry, Bicinchoninic acid assay, and Biuret assay), total amino acid content analysis, chromatographic, and radiolabelling methods. Among them, Kjeldahl nitrogen method is the most widely used method for protein quantification in samples. The Kjeldahl method was discovered in 1883 by the Danish chemist Johan Gustav Christoffer Thorsager Kjeldahl and used to determine the nitrogen and protein content. Publicly, it was made available on March 7, 1883, during the meeting held by Danish Chemical Society (Kemisk Forening) (7–9). Initially, this method was designed to aid in grain’s protein changes at the time of fermentation and germination in brewing industries (10). This method indirectly quantifies the total content of protein present in food by direct measurement of nitrogen (5, 11). Today, the Kjeldahl method of protein quantification is universally accepted and used in many laboratories for various food samples. It consists of three major steps, i.e., digestion of organic nitrogen-containing samples with sulfuric acid to ammonium sulfate, distillation of digested sample solution at an elevated temperature and pH to release ammonia which is trapped in boric acid solution, and finally titration of boric acid solution with standard acid (10). Although the method has various advantages, such as its universality, high precision, and reproducibility, it is time-consuming to prepare the sample (digestion) for the analysis (12). There is also a limitation w.r.t. analytical selectivity as it cannot differentiate between protein-based nitrogen and non-protein nitrogen. Some modifications in the standard Kjeldahl method have enhanced the versatility and decreased the procedural time for analyzing different samples simultaneously (11, 13–15). Lee and collaborators (16) compared the standard Kjeldahl method with three modified Kjeldahl procedures by the addition of salicylic acid prior to digestion, the pre-reduction of nitrate to ammonium using CrK(SO_4_)_2_, and the addition of phenyl-acetate to the standard digestion mixture. This last procedure yielded the best nitrogen measuring results in plant tissue, but the salicylic acid method performed better in the presence of water. Amin and Flowers (17) reported the use of salicylic acid dissolved in concentrated sulfuric acid to recover other nitro compounds. A Kjeldahl method guide (18) suggests the use of salicylic acid followed by sodium thiosulfate for nitrate reduction. Further modifications made by researchers have improved the digestion process. Studies have reported the use of ultrasound and microwave energy in the Kjeldahl procedure. Domini and collaborators (19) concluded that a combination of ultrasound and microwave energy in the digestion process improves its performance by reducing digestion time to 7 min compared to 30 min for the classical Kjeldahl. Ultrasound energy has been used to substitute the distillation system in classical Kjeldahl for a purge-and-trap system in order to stimulate a chemical reaction between the alkaline reagent and the digestion mixture (20). Although these methods provide rapid analysis of protein in the sample; however, they may require an additional instrument. Routinely, in laboratory analysis, there is a need for a fast, robust, accurate, and reliable method for estimating protein content.

The introduction of novel analytical approach in laboratory analysis requires comparison and validation with standard methods being followed for quality assurance. In the validation process, two important and complementary stages involves single- and inter-laboratory validations (21). Additionally, the protein’s quantitative analysis is important for accurate labeling of food as well as its quality control (22). Primarily used methods for protein nitrogen determination in food samples has also been utilized for determining various other nitrogen forms in plant materials, soils, wastewater matrices, and biological tissues (23, 24). To date, many methods have been developed for determining protein in food samples. However, Kjeldahl digestion and distillation ’method is most frequently used method (25). For successful analysis, proper handling of samples and sample preparation are the major steps, affecting the overall analysis time of the sample, thus need to redefine the analysis by developing novel methods.

Generally, the method initially requires a flour matrix for the digestion process that needs lots of time, especially when dealing with a large sample set. Mainly, homogenizing oilseeds is difficult due to high oil content; flour is often sticky, cleaning the homogenizer is complex, and carries a high risk of cross-contamination compared to cereals and pulses. During digestion, frothing occurs in the oilseed crops when using standard digestion mixture and to avoid this issue, we developed a modified digestion mixture. To accomplish quick protein quantification, we have used this modified digestion mixture to digest the whole grain of five oilseed crops and compared it with their flour matrix. The aim of this research was to evaluate and validate the developed novel analysis method to reduce the homogenization time, cleaning, and avoid the frothing. The method demonstrated that the protein content from whole grain of five tested oilseed crops shows 100% non-significant differences when compared with their flour matrices in both the digestion methods. Several statistical analyses such as descriptive statistics, correlations, paired *t*-test, reliability tests, and different types of plot are employed to validate the results. We hope that this method if utilized can help save sample processing time, costs, and minimize the risk of cross-contamination with accurate results.

## Materials and Methods

### Chemicals and Reagents

A high purity analytical grade chemicals such as methyl red, bromocresol green, lithium sulfate, sodium hydroxide pellets, hydrochloride salt, and selenium metal powder from Sisco Research Laboratory, India. Boric acid from Brunswick Scientific (United States), sulfuric acid from Qualigens (India), and hydrogen peroxide from Rankem Laboratory (India) was purchased.

### Plant Material and Preliminary Screening of Seeds

**FIGURE 1:**
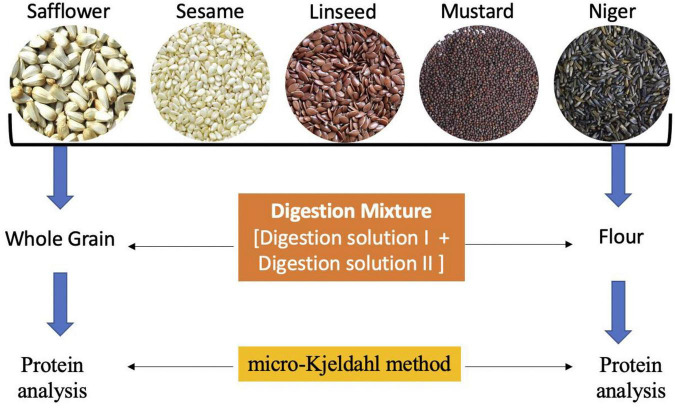
The modified sample preparation method with digestion mixture for protein analysis using Kejdhal method.

A total of five oilseed crops, i.e., safflower (12 accessions), sesame (10 accessions), linseed (10 accessions), mustard (10 accessions), and niger (10 accessions) were chosen for the study (Table 1) at ICAR-National Bureau of Plant Genetic Resources (NBPGR), New Delhi, India. Initially, the seeds were oven-dried to reduce the moisture content and grinded to fine flour using mixture grinder. Flour as well as seed samples were kept in air-tight sample containers for analysis of protein content. The samples were processed in two ways, first as whole-grain (WG1) and second as flour (FL1) samples using traditional digestion mixtures, and whole grain 2 (WG2) flour 2 (FL2) using modified digestion mixture (Figure 1). There were two main hypothesis of the study. First, The difference in estimated protein content in all the five oilseeds of whole grain (WG) and flour samples (FL) were non-significant with both digestion methods. The method of directly digesting whole grains can be used for small-seeded grains, as sample representativeness can be ensured; on the contrary, bold seeds limit sample representativeness and will reduce grinding time and fasten the protein quantification. Second, the modified digestion mixture is less time consuming over the traditional digestion mixture. The traditional digestion mixture contains sulfuric acid (specific gravity 1.84), nitrogen-free catalyst mixture (K_2_SO_4_ and CuSO_4_), whereas the modified digestion mixture protocol is given below.

**TABLE 1:** Accession number and protein concentration of all the five oilseed crops for flour and whole-grain for both digestion mixtures.

Sr. no.	Accession no.	Traditional DM		Modified DM	
		WG1	FL1	WG2	FL2
**Safflower**					
1	NIC594327	14.78	15.01	15.26	15.28
2	IC138882	17.33	16.91	16.99	16.95
3	NIC7094	18.6	17.99	18.35	18.39
4	IC96004	14.38	14.86	14.21	14.62
5	IC95994	15.39	15.68	15.10	14.99
6	PI250202	18.1	17.89	17.99	18.01
7	IC305162	16.3	16.46	16.06	15.99
8	LSRM-14-38	16.2	15.68	15.89	16.01
9	IC95966	19.35	19.87	18.96	19.35
10	IC11122	12.39	12.45	12.45	12.25
11	IC95980	19.25	18.98	19.02	18.99
12	IC96017	19.36	18.99	18.87	18.97
**Sesame**					
13	IC0430504	21.94	22.07	21.67	21.83
14	IC0430512	19.37	18.97	19.87	19.01
15	IC0430614	23.28	23.68	23.50	23.68
16	IC0500438	26.12	25.87	26.10	25.97
17	IC0500837	23.99	24.22	24.01	24.22
18	IC0501037	23.47	22.98	23.35	23.06
19	IC0510962	21.22	21.79	21.20	20.97
20	IC0510964	17.71	17.61	17.57	17.72
21	IC0510975	18.19	18.40	18.23	18.32
22	IC0510980	19.47	18.93	19.34	18.9
**Mustard**					
23	IC491078	25.14	25.24	24.89	24.99
24	IC122369	23.83	23.63	24.03	23.89
25	IC122423	23.72	23.11	23.78	23.67
26	IC122032	24.14	23.93	24.24	24.45
27	IC10976	25.87	25.34	25.67	25.45
28	IC11037	22.3	22.31	22.42	23.01
29	IC122441	24	24.25	23.98	23.78
30	IC73236	26.5	25.89	26.38	26.43
31	IC491280	23.73	23.16	23.59	23.19
32	IC491181	27.4	26.99	27.57	26.89
**Linseed**					
33	IC0096509	19.13	19.37	19.72	20.09
34	IC0096510	19.34	18.97	19.11	18.99
35	IC0096520	21.1	20.98	20.97	21.01
36	IC0096524	19.13	19.25	18.99	18.94
37	IC0096525	20.6	20.31	20.79	20.88
38	IC0096526	17.71	17.05	17.80	16.89
39	IC0096527	17.69	17.71	17.67	17.01
40	IC0096528	19.34	18.79	19.27	18.99
41	IC0096529	18.59	18.51	18.68	18.71
42	IC0096530	19.11	18.97	19.01	18.99
**Niger**					
43	IC305116	20.3	20.49	19.45	19.34
44	IC412911	19.01	19.03	18.97	18.89
45	IC510922	15.06	14.90	15.1	15.3
46	IC545083	22.01	21.67	21.89	22
47	IC552739	17.49	17.54	17.56	17.7
48	IC552811	15.21	15.76	15.1	15.6
49	IC564781	18.04	17.78	17.89	17.95
50	IC565448	20.12	20.21	20.32	20.41
51	IC617173	16.97	16.85	16.96	17.06
52	IC618584	20.02	21.26	20.00	20.09

*DM, digestion mixture*

#### Modified Protocol Development

##### Digestion Solution I

Briefly, 1.6 g of selenium powder was added to 450 mL of concentrated H_2_SO_4_, and the mixture was heated till H_2_SO_4_ became pale yellow. The sulfuric acid-Se solution was cooled down to room temperature and, after that placed in a deep freezer at –20°C.

##### Digestion Solution II

A 14.0 g of lithium sulfate or sodium sulfate was dissolved in 350 mL of hydrogen peroxide solution (30% AR grade), and the solution was cooled in an ice bath.

##### Digestion Mixture

To the chilled hydrogen peroxide solution (Digestion solution II), which was kept in an ice bath, the chilled sulfuric acid solution (Digestion solution I) was added slowly. The digestion mixture was thus prepared is fit for use till one week if stored at 5–8°C in the refrigerator and for one month at –20°C in deep freezer without any loss of activity.

### Protein Analysis

Analytical micro-Kjeldahl method for determining crude protein was conducted by estimating total nitrogen (11, 12) using FOSS autoanalyzer (Model 2300 Kjeltec unit).

### Statistical Analysis

The results were analyzed statistically using univariate and multivariate statistics, two-tailed Pearson correlations at a significance level of 1 and 5%, reliability test, and paired *t*-test and different types of plot analysis have been conducted (26–32).

The analysis was performed using SPSS 17 (International Business Machines, United States).

## Results

The present study was designed to modify the standard Kjeldahl method to quantify the protein content in five oilseed crops. Sample processing/preparation is the first major step required to successfully analyze chemical content. By directly digesting the whole grain of oilseed crops, protein quantification was accomplished quickly, and the results are comparable. The validation of this novel analysis demonstrated that the protein content from whole grain of oilseed crops showed 100% non-significant results compared with the flour matrix.

### Statistical Analysis

#### Descriptive Statistics

**FIGURE 2:**
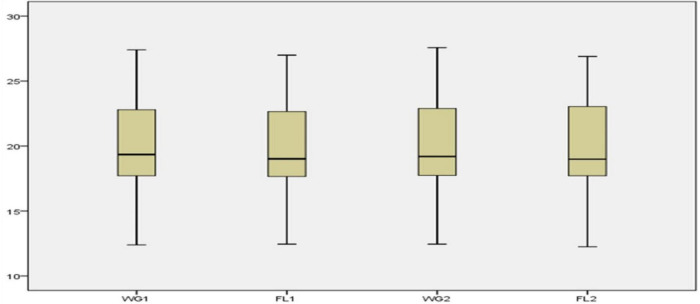
Box plots of WG1, FL1, WG2, and FL2.

By using a total of 52 accessions of five oilseed crops, various descriptive statistical analyses were performed to validate the findings. Four categories were made, including whole grain (WG1) and flour (FL1) with traditional digestion mixture and whole grain (WG2) and flour (FL2) with modified digestion mixture. For each sample, the statistical analysis such as range, mean, standard deviation, variance, skewness, and kurtosis was calculated (Table 2). The Box-plot represents the locality, spread, and skewness groups of numerical data through their quartiles (33). For each category, there is no major difference in the mean, standard deviation, variance, and skewness values for the flour and whole grain samples under traditional and modified digestion mixture (Figure 2). It showed that the developed method is robust and provides meaningful results without any variance in the data compared to the traditional method.

**TABLE 2:** Descriptive statistical analysis showing range, mean, standard deviation, variance, skewness, and kurtosis for flour and whole-grain of the five oilseed crops.

Descriptive statistics									
	*N*	Range	Min	Max	Mean ± SE	Std. Dev	Variance	Skewness	Kurtosis
WG1	52	15.01	12.39	27.40	19.97 ± 0.47	3.42	11.73	0.21 ± 0.33	–0.43 ± 0.65
FL1	52	14.54	12.45	26.99	19.89 ± 0.46	3.35	11.25	0.20 ± 0.33	–0.58 ± 0.65
WG2	52	15.12	12.45	27.57	19.91 ± 0.47	3.44	11.87	0.24 ± 0.33	–0.47 ± 0.65
FL2	52	14.64	12.25	26.89	19.88 ± 0.47	3.41	11.64	0.22 ± 0.33	–0.56 ± 0.65

#### Two-Tailed Pearson Correlation

The Sig (2-tailed) Pearson correlation of *p*-value describes significant correlation at a particular level. Smaller the *p*-value, significant is the correlation (26). By using a total of 52 accessions of five oilseed crops two-tailed Pearson correlation was conducted for all the four categories, i.e., WG1, FL1, WG2, and FL2 in order to identify the correlations between the flour and whole grain protein as well as for both the digestion mixtures. Correlation is significant at the (0.01 level) (2-tailed), i.e., the value will be considered significant if it lies between 0.001 and 0.010. For each sample the correlation analysis has been done, and showed the 100% significant result in whole grain and flour samples (Table 3). We can easily identify the bivariate Pearson correlation coefficient (with a two-tailed test of significance) using paired samples correlations for each pair of entered variables, i.e., pair 1 (WG1 and FL1), pair 2 (WG2 and FL2), pair 3 (WG1 and WG2), and pair 4 (FL1 and FL2). Therefore, our result supports both the hypothesis that there is no significant difference in the protein quantity for samples (flour and whole- grain) treated with traditional or modified digestion mixtures.

**TABLE 3:** Two-tailed Pearson correlation analysis in flour and whole-grain digested with traditional and modified digestion mixtures.

	FL1	WG2	FL2
WG1	0.994** *p* = 0.00	0.998** *p* = 0.00	0.994** *p* = 0.00
FL1		0.992** *p* = 0.00	0.993** *p* = 0.00
WG2			0.996** *p* = 0.00

**Correlation is significant at the 0.01 level (2-tailed).

#### Reliability Tests

The reliability tests played a significant role in quantitative research and are considered instruments for measurement. Reliability provides consistency and accuracy in a given method (27). By using a total of 52 accessions of oilseed crops, reliability tests have been conducted (Tables 4, 5A–C) taking all variables as scale, “Strict parallel model” to test the goodness of fit model, analysis of variance (ANOVA) with Tukey’s Test for Non-additivity, Hotelling’s *T*-squared test, and inter-class correlations.

**TABLE 4:** Tests of normality.

	Kolmogorov–Smirnov^a^			Shapiro–Wilk		
	Statistic	*df*	Sig.	Statistic	*df*	Sig.
WG1	0.136	52	0.018	0.976	52	0.361
FL1	0.121	52	0.055	0.976	52	0.379
WG2	0.112	52	0.123	0.975	52	0.343
FL2	0.140	52	0.013	0.971	52	0.226

*^a^Lilliefors significance correction.*

**TABLE 5A:** Hotelling’s *T*-squared test.

Hotelling’s *T*-squared	*F*	*df*1	*df*2	Sig
5.206	1.667	3	49	0.186

**TABLE 5B:** ANOVA with Tukey’s test for non-additivity.

		Sum of squares		*df*	Mean square	*F*	Sig
Between people			2361.887	51	46.312		
Within people	Between items		0.260	3	0.087	1.309	0.274
	Residual	Non-additivity	0.059^a^	1	0.059	0.884	0.349
		Balance	10.073	152	0.066		
		Total	10.131	153	0.066		
	Total		10.391	156	0.067		
Total			2372.279	207	11.460		

*Grand mean = 19.9192.*

*^a^Tukey’s estimate of power to which observations must be raised to achieve additivity = –1.806.*

**TABLE 5C:** Intraclass correlation coefficient.

	Intraclass correlation^b^	95% confidence interval		*F* test with true value 0			
		Lower bound	Upper bound	Value	*df*1	*df*2	Sig
Single measures	0.994^a^	0.991	0.996	699.389	51	153	0.000
Average measures	0.999	0.998	0.999	699.389	51	153	0.000

*Two-way random effects model where both people effects and measures effects are random.*

*^a^The estimator is the same, whether the interaction effect is present or not.*

*^b^Type A intraclass correlation coefficients using an absolute agreement definition.*

Based on the statistical analyses, the reliability of the scale was 0.999, and the common Inter-Item correlation was 0.994, which is considered as “the best” and well supported our hypothesis as well.

The Intraclass correlation coefficient (ICC) is utilized in assessing agreement if there are two or more independent raters (which should be independent). In this, the resultant outcome is determined at a continuous level. It is considered as the more powerful reliability test due to utilization of continuous measurement (Table 5C).

##### Test for Goodness of Fit Model

A chi-square test is used to test the relationship between two categorical variables, and is generally known as the test for goodness of fit model. It generally shows the difference between observed counts and expected counts of the dataset if no relationship is found in the population (28) McHugh. The chi-square test value for all the 52 accessions of oilseed crops was 38.278 with an 11 degree of freedom at *p* < 0.000 significance level under the strictly parallel model assumption. The log of the determinant of an unconstrained matrix was –5.004, and for constrained matrix, it was –4.234. The results strongly favor both hypothesis.

**FIGURE 3:**
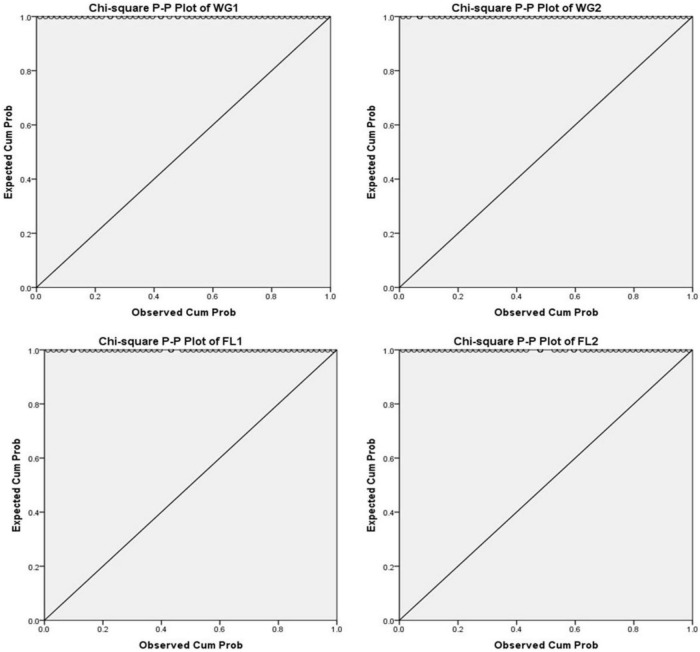
Chi-square P-P plots of WG1, WG2, FL1, and FL2.

**FIGURE 4:**
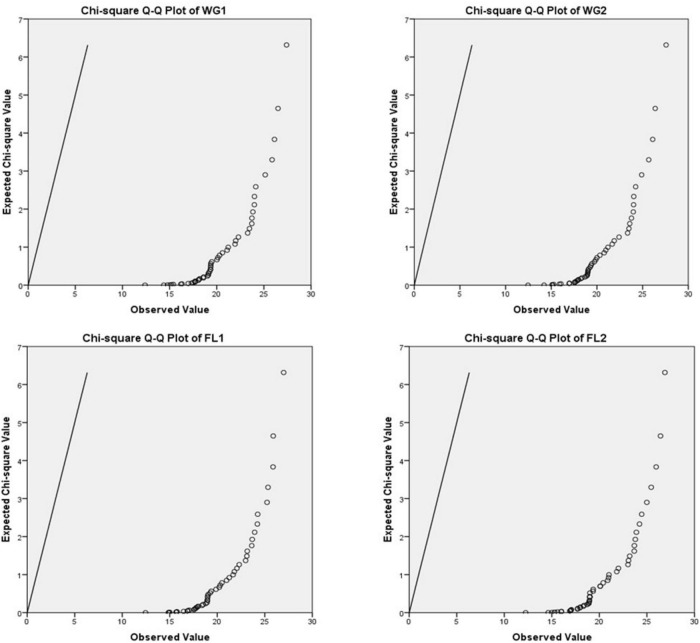
Chi-square Q-Q plots of WG1, WG2, FL1, and FL2.

**FIGURE 5:**
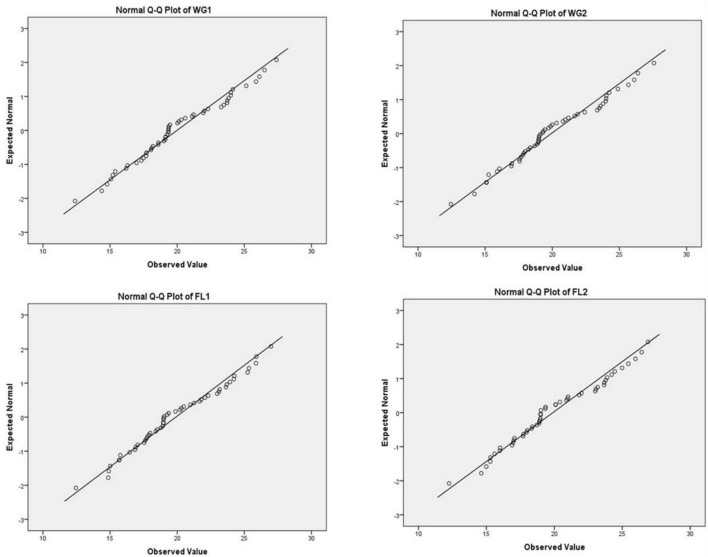
Normal Q-Q plots of WG1, WG2, FL1, and FL2.

A P-P plot is generally used for comparing the empirical cumulative distribution function of data to that with specified theoretical cumulative distribution function *F* (⋅). In contrast, a Q-Q plot compares quantiles of the distributed dataset with the standardized theoretical distribution quantiles from specific family distributions. The P-P requires the location and scale parameters to visualize the linear pattern intercept and slope, whereas Q-Q plot does not require these parameters (31, 32; Figures 3–5).

According to the null hypothesis, the expected and observed protein concentration is the same across all the treatments (whole grain and flour) for both the digestion mixtures. For plotting P-P plots, Bloom’s Fractional Rank Estimation Method is applied (Figure 3). The model indicates that protein concentration is significantly associated with the treatments (*p* < 0.001). The P-P plots showed the observed proportion of protein concentration in the data and the expected proportion, as predicted by the model. Ideally, all the points fall on the diagonal line producing the best fit and supporting the null hypothesis.

For plotting Q-Q plots (Figures 4, 5), Kolmogorov–Smirnov and Shapiro–Wilk tests were applied. The higher *p*-value suggested that the differences in protein concentration of the whole grain and flour; and for both the digestion mixtures are non-significant and strongly supporting the proposed hypothesis (Table 4). The Q-Q plots for WG1 and FL1, and WG2 and FL2 are similar and there is no significant deviation for the treatments (Figures 4, 5).

#### Hotelling’s *T*-Squared Test

In statistical analysis, particularly in hypothesis testing, one of the methods developed by Harold Hotelling, known as the Hotelling’s *T*-squared distribution (*T*^2^), is a multivariate probability distribution tightly related to the *F*-distribution and a generalization of Student’s *t*-test (30). Results showed that the Hotelling’s *T*-Squared value is much higher (5.206) than the *F*-value (1.667) with a significance level of 0.186 (Table 5A), hence supporting the hypothesis.

#### Analysis of Variance With Tukey’s Test for Non-additivity

Tukey’s test of non-additivity shows an interaction between different factors with no replication. Hence, it has been utilized to test the interaction between the treatment and block factors in a randomized block design. Each block shows the interactions involving different magnitudes but not different directions of treatment effects (29; Table 5B).

Interclass correlation coefficient at 95% confidence interval was 0.994 for single measures and 0.994 for average measures (Table 5C), providing a robust correlation and supporting both the hypothesis.

#### Paired *T*-Test

The paired sample test, commonly known as the dependent sample test, is a statistical method utilized to determine the mean difference between two datasets and is thought to be zero. Each entity is measured two times and observed as a pair of items. Generally, it gives the hypothesis test results. In this research, four pairs, i.e., pair 1 (WG1 and FL1), pair 2 (WG2 and FL2), pair 3 (WG1 and WG2), pair 4 (FL1 and FL2) has been tested. For each pair, the differences in the mean and standard deviation are near to zero (Table 6A). The t-score ranges from 0.15 to 1.73, for all the four categories under investigation and supports the null hypothesis (Table 6B).

**TABLE 6A:** Paired sample statistics and paired samples test showing mean difference and standard deviation.

Paired samples statistics									
		Paired differences							
		Mean	Std. dev	Std. error mean	95% confidence interval of the difference		*t*-score	*df*	Sig. (2-tailed)
					Lower	Upper			
Pair 1	WG1-L1	0.08 ± 0.05	0.38	0.05	–0.02	0.18	1.52	51	0.13
Pair 2	WG2-L2	0.03 ± 0.04	0.30	0.04	–0.05	0.11	0.79	51	0.43
Pair 3	WG1-G2	0.05 ± 0.03	0.24	0.03	–0.00	0.12	1.73	51	0.09
Pair 4	FL1-FL2	0.00 ± 0.05	0.41	0.05	–0.10	0.12	0.15	51	0.87

**TABLE 6B:** Paired sample statistics and correlations showing mean, standard deviation, and correlations.

Paired samples statistics and correlations							
		Mean	*N*	Std. deviation	Std. error mean	Correlation	Sig.
Pair 1	WG1	19.97	52	3.42	0.47	0.994	0.000
	FL1	19.89	52	3.35	0.46		
Pair 2	WG2	19.91	52	3.44	0.47	0.996	0.000
	FL2	19.88	52	3.41	0.47		
Pair 3	WG1	19.97	52	3.42	0.47	0.998	0.000
	WG2	19.91	52	3.44	0.47		
Pair 4	FL1	19.89	52	3.35	0.46	0.993	0.000
	FL2	19.88	52	3.41	0.47		

## Discussion

The health benefits of plant-based protein from different sources are recognized and much appreciated, particularly for the low cost of production and carbon footprint (34–36). The Alternatives to legumes are needed to cater to the increasing demand for plant based protein. The high protein content (15–50%) is reported in different oilseeds, making them ideal sources. Kjeldahl method is most commonly used for determining protein content in the food samples. As a standard practice, samples are ground before digestion. However, oilseeds often form cake while grinding due to high oil content; thus, cleaning grinding mills is challenging and increases the risk of sample cross contamination. The introduction of novel analytical approach in laboratory analysis requires validation of previous methods for assurance of its quality. Some modifications in the standard Kjeldahl method have enhanced the versatility and decreased the procedural time for analyzing different samples (16, 18–20). Hydrogen peroxide is added to digest the samples with high oil content to prevent foaming and aid rapid digestion. However, proper sample handling, their sampling, and analytical procedure followed are essential for accurate and precise analysis. In our research, we directly digested the whole grain of five different oilseed crops using two different digestion methods to accomplish quick protein quantification. And with the modification in digestion mixture and catalysts the process of protein quantification speeds up. There were two hypotheses in this study. First, for small-seeded oilseeds, whole grain (WG) gives equally precise results as with flour samples (FL). Therefore, using WG will speed up the protein quantification method. Second, the modified digestion mixture and traditional digestion mixture are equally effective and have no influence on nitrogen recovery. The descriptive statistical analysis shows that no major difference in the values of mean, standard deviation, variance, and skewness for the flour as well as whole grain samples. With paired sample test, which is used for determination of mean difference between two datasets, it has been found that the difference in the mean, as well as standard deviation is near to zero for each pair, i.e., pair 1 (WG1 and FL1), pair 2 (WG2 and FL2), pair 3 (WG1 and WG2), and pair 4 (FL1 and FL2). The Sig (2-tailed) Pearson correlation describes 100% significant result in whole grain and flour samples. Reliability test based on the results from 52 accessions belonging to five different oilseeds, demonstrated consistency of the results. The value of chi-square test for all the 52 accessions of oilseed crops was found to be 38.278 with 11 degree of freedom at zero significance, which shows the relationship between two categorical variables. Hotelling’s *T*-squared distribution (T2), a multivariate probability distribution, which is tightly related to the F-distribution and a generalization of ‘Student’s *t*-test, shows the significance of 0.186. Tukey’s test of non-additivity is a test that shows an interaction between different factors with no replication. Thus the validation of this novel analysis/modified approach of using whole grain in small-seeded oilseeds demonstrated non-significant differences with their respective flour samples.

## Conclusion

For the determination of “food”s protein quantity, it is very essential to standardized analytical methods and shortens the overall analysis time. The correct quantification as well as determination of content of protein is important, and hence in the present study we analyze and compare the modification of Kjeldahl method for determination of protein content in five oilseed crops. Here, we directly digested whole grain of five oilseed crops instead of making flour to reduce processessing time and accomplish quick protein quantification. Further, to cope up with oil frothing during digestion time, we developed a modified digestion mixture. On the basis of validation parameters analyzed, it can be concluded that the protein content from whole grain of oilseed crops shows 100% non-significant results compared with the flour matrix indicating that there is no difference in the protein content if we digest the whole rain rather than that of the flour. Statistical analyses such as paired *T*-test, Pearson correlation, a range of reliability tests, and different types of plot analysis showed similar results in both flour and whole grain (reduce procedural time) for both the digestion mixtures. Hence, this method will help saving sample processing time, costs, and minimize the risk of cross-contamination with accurate results. This will enable a large data set to be finished in a very less duration of time.

## Data Availability Statement

The original contributions presented in the study are included in the article/supplementary material, further inquiries can be directed to the corresponding authors.

## Author Contributions

SL conceived the idea and contributed to experimentation and manuscript drafting. RB standardized digestion methods and proofread the manuscript. RB and SL contributed to statistical analysis. RY and JR provided diverse accessions of oilseeds. AK and SK contributed to resources, editing, and formal analysis. All authors contributed to the article and approved the submitted version.

## Conflict of Interest

The authors declare that the research was conducted in the absence of any commercial or financial relationships that could be construed as a potential conflict of interest.

## Publisher’s Note

All claims expressed in this article are solely those of the authors and do not necessarily represent those of their affiliated organizations, or those of the publisher, the editors and the reviewers. Any product that may be evaluated in this article, or claim that may be made by its manufacturer, is not guaranteed or endorsed by the publisher.
